# Oxidation of l-leucine amino acid initiated by hydroxyl radical: are transition metal ions an enhancement factor?

**DOI:** 10.1098/rsos.220316

**Published:** 2022-09-14

**Authors:** Dinh Hieu Truong, Thi Chinh Ngo, Thi Huong Lan Nguyen, Duy Quang Dao

**Affiliations:** ^1^ Institute of Research and Development, Duy Tan University, Da Nang 550000, Vietnam; ^2^ Faculty of Natural Sciences, Duy Tan University, Da Nang 550000, Vietnam; ^3^ Faculty of Pharmacy, Duy Tan University, Da Nang 550000, Vietnam

**Keywords:** DFT, l-leucine, iron complexes, copper complexes, antioxidant, pro-oxidant

## Abstract

Hydroxyl radical (HO·) formation initiated by the Fenton-type reactions of Fe and Cu complexes of l-leucine (Leu) amino acid as well as its oxidation reaction by HO· was computationally investigated by using the density functional theory method at the M05-2X/6-311++G(3df,2pd)//M05-2X/6-311++G(d,p) level of theory in the aqueous phase. The results showed that dipole-salt is the main form of Leu in the physiological condition. Leu exhibits high chelating potential towards both Fe(III)/Fe(II) and Cu(II)/Cu(I) ions with the most favourable coordinating positions at two oxygen atoms of the –COO functional group. Furthermore, the Leu-ions complexes show a high risk of HO· formation via Fenton-like reactions, especially when ascorbate anion exists in the environment as a reducing agent. Finally, the oxidation reaction of l-leucine by HO· demonstrated a relatively high overall apparent reaction rate, *k*_overall_, being 1.18 × 10^9^ M^−1^ s^−1^, in which formal hydrogen transfer reactions of the dipole-salt form occur as the primary mechanism. Consequently, the Leu oxidation by HO· radical can be promoted by the Fenton reaction enhancement of its transition metal complexes.

## 1. Introduction

Amino acids consist of biomolecules containing the amino and carboxyl functional groups [1–5]. Until 2020, more than 500 amino acid compounds have been found in nature [1], among which only 20 α-amino acids appear in the genetic code [2]. They are also the building block of protein and several substances like neurotransmitters, hormones and nucleic acids [3]. In the human body, only 0.5 to 1.0% of the total amino acids are present as free amino acids in plasma or the intracellular and extracellular spaces [4]. In free form, natural α-amino acids, except glycine, commonly exist in L-configuration.

Amino acids have already shown the complexation ability towards different transition metal ions such as Fe(III), Fe(II), Cu(II) and Cu(I), owing to their carboxyl and amino functional groups [6–9]. In biological systems, these complexes play essential roles in electron transfer, catalysis, structural support and protein folding/unfolding [6,7,10]. Therefore, they have been attracting many scientists in both experimental and theoretical fields [6–8]. Elius Hossain *et al*. studied the interaction between phenylalanine (Phe) and five transition metal ions, including Mn(II), Co(II), Ni(II), Cu(II) and Zn(II) [7] at the B3LYP/SDD//6-31G(d) level of theory. Experimental and computed infrared results demonstrated a significant change in vibrational frequencies of metal-Phe complexes compared with the free ligand. In addition, density functional theory (DFT) results showed that Zn complexes have existed in tetrahedral structures, whereas the primary forms of other complexes are square planar. Moreover, the binding energies of these complexes vary from −304.9 to −198.5 kcal mol^−1^, with the most stable complexes being Ni(Phe)_2_.

**Figure 1.**
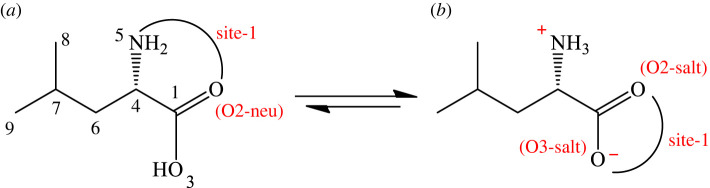
Two-dimensional structure of l-leucine in two forms: neutral (*a*) and dipole-salt (*b*).

On the other hand, free amino acids may exhibit pro-oxidant activities leading to the formation of reactive hydroxyl radical (HO·) Ka *et al*. [11] studied the antioxidant and pro-oxidant properties of several amino acids by using *in vitro* assay and oil-in-water (O/W) emulsions under riboflavin (RF) photosensitization. Their results showed that cysteine has the highest antioxidant properties, followed by tryptophan and tyrosine. However, tyrosine inhibits lipid oxidation in O/W emulsions under RF photosensitization, whereas tryptophan acts as a pro-oxidant. Besides, amino acids have also proven their pro-oxidant risks when transition metal ions are available in the environment. Milach *et al*. [12] investigated the pro-oxidant properties of amino acids and their derivatives in the presence of Fe(II) and Cu(II) ions and illustrated the pro-oxidant activity of cysteine in the Fe(II)/EDTA system.

l(-)-Leucine (Leu), a branched-chain amino acid, is an essential amino acid that cannot be synthesized from scratch by the organism fast enough to supply its demand, and it must therefore be obtained from the diet [3,13–15]. It is commonly found in foods containing protein, such as soy products, beans and other legumes. In protein, the content of l-leucine varies from 5 to 10% [14], which plays a crucial role in the protein synthesis stimulation in muscle [15]. Like other α-amino acids, which have only one amino and one carboxyl functional group, the free l-leucine exists in two forms: neutral and dipole-salt (figure 1) [5–7]. The latter is identified as the primary form of Leu in the physiological environment (pH = 7.4) [14]. The oxidation rate of Leu is determined to be higher than that of other branched-chain amino acids, for example, isoleucine and valine [16]. Furthermore, Medina *et al.* [17] theoretically investigated the reactions occurring between Leu and several radicals, including ·N_3_, Cl, OH, ·OCH_3_, ·OCH_2_Cl, and ·OCHCl_2_, ·OOH, ·OOCH_3_, ·OOCH_2_Cl, ·OOCHCl_2_, ·OOCCl_3_ and ·OOCHCH_2_. Their results illustrated that the prominent reactive position of these reactions is found at the gamma site (C7-H, figure 1) for the reactions towards ·N_3_, ·OOCCl_3_, ·OCH_3_, ·OCH_2_Cl, and ·OCHCl_2_ radicals, with the rate constants varying from 3.24 × 10^4^ to 8.87 × 10^8^ M^−1^s^−1^. The Leu reacts with the ·Cl, ·OH and ·OCCl_3_ radicals at the beta, gamma and delta positions at reaction rates close to the diffusion limit (approx. 10^9^ M^−1^s^−1^). Although the properties of l-leucine have widely been studied from different points of view, its chelating ability towards transition metal ions and its oxidation by free radicals are still limited. Besides, the relation between the complexation properties and the pro-oxidant risk of Leu amino acid should be clarified.

Therefore, in this study, we firstly focused on the chelating ability of l-leucine towards transition metal ions, including Fe(II), Fe(III), Cu(I) and Cu(II) in the aqueous phase. The standard enthalpies (Δ_r_*H*^0^) and Gibbs free energies (Δ_r_*G*^0^) of chelating reactions were calculated to evaluate the stability of the formed complexes. The reduction reactions of these complexes by two reducing agents, namely superoxide anion (O_2_·^–^) and ascorbate anion (Asc^–^), were then taken into account to estimate the HO· radical formation risks via Fenton-type reactions. Finally, the kinetics of Leu amino acid oxidation reactions by the formed HO· radical via hydrogen transfer reactions were studied.

## 2. Computational method

All geometry optimizations and vibrational frequency calculations for reactants, transition states (TSs), pre-reactive complexes, post-reactive complexes and products were performed by Gaussian 16 Rev. A.03 package [18] in the aqueous phase using M05-2X functional [19] and 6-311++G(d,p) basis set. Single-point calculations then improved the accuracy of the energy values at the M05-2X/6-311++G(3df,2pd) level of theory. The influence of the aqueous media was mimicked by combining the implicit solvation model based on density (SMD) [20] and the polarizable continuum model (PCM). While the PCM is one of the most used and reliable continuum solvation procedures, the SMD corrects CDS (i.e. cavitation, dispersion and solvent structure effects). As a result, the combination of PCM and SMD has been recommended in the QM-ORSA [21] approaches for kinetics of free radical—molecules in solvents. It has been widely used by several works in the literature [22]. The structures of [Fe(H_2_O)_6_]^3+^, [Fe(H_2_O)_6_]^2+^, [Cu(H_2_O)_4_]^2+^ and [Cu(H_2_O)_4_]^+^ were employed as recommended by previous works [23–26]. In this study, the high-spin states of hydrated Fe(II) and Fe(III) ions being 5 and 6, respectively, were used as recommended by several works in the literature [25–30].

The complexation reaction between metallic ions and l-leucine (Leu) was described as follows:

2.1 $${\left[M{\left(H_{2}O\right)}_{m}\right]}^{x+}+\mathrm{Leu}\to {\left[M{\left(H_{2}O\right)}_{m-n}\mathrm{Leu}\right]}^{x+}+nH_{2}O,$$

where the values *n* = 1 and 2 represent the mono- or bidentate complexes, respectively. M denotes the studied metals, including Fe and Cu, whereas *m* is the number of water molecules in the metal ions (*m* = 6 and 4 for Fe and Cu ions, respectively). *x* is the positive charge of metallic ions (*x* = +2 or +3 for Fe and *x* = +1 or +2 for Cu). Thus, the standard Δ_r_*H*^0^ and Δ_r_*G*^0^ values for the complexation reactions were calculated by the following equations (2.2) and (2.3):

2.2 $$\Delta_{r}H^{0}=H({\left[M{\left(H_{2}O\right)}_{m-n}\mathrm{Leu}\right]}^{x+})+nH(H_{2}O)\text{–}H(\mathrm{Leu})\text{–}H({\left[M{\left(H_{2}O\right)}_{m}\right]}^{x+});$$

2.3 $$\Delta_{r}G^{0}=G({\left[M{\left(H_{2}O\right)}_{m-n}\mathrm{Leu}\right]}^{x+})+nG(H_{2}O)\text{–}G(\mathrm{Leu})\text{–}G({\left[M{\left(H_{2}O\right)}_{m}\right]}^{x+}).\;$$

To characterize the complexation reactions, the equilibrium constants, *K_f_* commonly called the formation constants, were also calculated by equation (2.4) [25,28,31].

2.4 $$K_{f}=e^{-(\Delta_{r}G^{0}/RT)}.\;$$

Pro-oxidant risks of Leu were determined via the reduction reactions of Fe(III)-to-Fe(II) and Cu(II)-to-Cu(I) complexes by two reducing agents, including superoxide anion (O_2_·^–^) and ascorbate anion (Asc^−^). These initiative reactions that lead to the formation of Fe(II) and Cu(I) ions involved in Fenton-like reactions producing reactive HO· [32] occurred as the following reactions (2.5, 2.6):

2.5 $${\left[M{\left(H_{2}O\right)}_{m-n}\mathrm{Leu}\right]}^{y+}+O_{2}^{\cdot -}\to {\left[M{\left(H_{2}O\right)}_{m-n}\mathrm{Leu}\right]}^{(y-1)+}+O_{2}$$

2.6 $${\left[M{\left(H_{2}O\right)}_{m-n}\mathrm{Leu}\right]}^{y+}+{\mathrm{Asc}}^{\text{–}}\to {\left[M{\left(H_{2}O\right)}_{m-n}\mathrm{Leu}\right]}^{(y-1)+}+{\mathrm{Asc}}^{\cdot },$$

where *y* = +3 and +2 values are the charges of iron and copper complexes, respectively.

The standard enthalpies (Δ_r_*H*^0^) and Gibbs free energies (Δ_r_*G*^0^) of reactions (2.5) and (2.6) were calculated as equations (2.7)/(2.8) and (2.9)/(2.10).

For superoxide anion radical (O_2_·^–^)

2.7 $$\Delta_{r}H^{o}=H({\left[M{\left(H_{2}O\right)}_{m-n}\mathrm{Leu}\right]}^{(y-1)+})+H(O_{2})\text{–}H({\left[M{\left(H_{2}O\right)}_{m-n}\mathrm{Leu}\right]}^{y+})\text{–}H(O_{2}^{\cdot -});$$

2.8 $$\Delta_{r}G^{o}=G({\left[M{\left(H_{2}O\right)}_{m-n}\mathrm{Leu}\right]}^{(y-1)+})+G(O_{2})\text{–}G({\left[M{\left(H_{2}O\right)}_{m-n}\mathrm{Leu}\right]}^{y+})\text{–}G(O_{2}^{\cdot -}).\;$$

For ascobate anion (Asc^–^)

2.9 $$\Delta_{r}H^{o}=H({\left[M{\left(H_{2}O\right)}_{m-n}\mathrm{Leu}\right]}^{(y-1)+})+H({\mathrm{Asc}}^{\cdot })\text{–}H({\left[M{\left(H_{2}O\right)}_{m-n}\mathrm{Leu}\right]}^{y+})\text{–}H({\mathrm{Asc}}^{\text{–}});$$

2.10 $$\Delta_{r}G^{o}=G({\left[M{\left(H_{2}O\right)}_{m-n}\mathrm{Leu}\right]}^{(y-1)+})+G({\mathrm{Asc}}^{\cdot })\text{–}G({\left[M{\left(H_{2}O\right)}_{m-n}\mathrm{Leu}\right]}^{y+})\text{–}G({\mathrm{Asc}}^{\text{–}}).\;$$

The obtained Δ_r_*H*^0^ and Δ_r_*G*^0^ values for the redox reactions of the Leu-ions complexes (2.5, 2.6) were then compared with the ones of hydrated iron and copper ions (2.11, 2.12):

2.11 $${\left[M{\left(H_{2}O\right)}_{m}\right]}^{y+}+O_{2}^{\cdot -}\to {\left[M{\left(H_{2}O\right)}_{m}\right]}^{(y-1)+}+O_{2}$$

2.12 $${\left[M{\left(H_{2}O\right)}_{m}\right]}^{y+}+{\mathrm{Asc}}^{\text{–}}\to {\left[M{\left(H_{2}O\right)}_{m}\right]}^{(y-1)+}+{\mathrm{Asc}}^{\cdot }.$$

The rate constants of the redox reactions and the oxidation reaction of Leu by HO· radical were calculated using the conventional TS theory approach. The thermodynamic equivalent was employed in KiSThelP as the following equation (2.13) [33]:

2.13 $$k^{\mathrm{TST}}(T)=\sigma \frac{k_{B}T}{h}{\left(\frac{RT}{P^{0}}\right)}^{\Delta n}e^{-\Delta G^{0,\neq }(T)/RT},$$

where *σ* is the reaction symmetry number or the reaction path degeneracy; *k*_B_, *h* and *R* are the Boltzmann, Planck and molar gas constants, respectively; *T* is the temperature of the system, and Δ*G*^‡^ is the Gibbs free energy of activation; Δ*n* = 1 or 0 for bimolecular and unimolecular reactions, respectively.

For the formal hydrogen transfer (FHT) reactions, *ΔG*^‡^ was determined by the differences in Gibbs energy between TS and reactants, while the value for the SET process was calculated using Marcus's theory [34,35].

2.14 $$\Delta G^{\text{‡}}=\frac{\lambda }{4}{\left(1+\frac{\Delta G_{\mathrm{SET}}^{0}}{\lambda }\right)}^{2},$$

where *λ* is the nuclear reorganization energy and $\Delta G_{\mathrm{SET}}^{0}$ is the Gibbs free energy of the SET reaction. The *λ* value is the difference between $\Delta G_{\mathrm{SET}}^{0}$ and $\Delta E_{\mathrm{SET}}$ that is the non-adiabatic energy between reactants and products (equation (2.15)).

2.15 $$\lambda =\Delta E_{\mathrm{SET}}-\Delta G_{\mathrm{SET}}^{0}.$$

In the KiSThelP, the Wigner correction, *χ*(*T*), is applied to acquire the tunnelling correction factor for all the elementary reactions [33,36,37]. This factor was determined based on the imaginary frequency lm(*v^‡^*) of the TSs as follows [33]:

2.16 $$\chi (T)=1+\frac{1}{24}\left(\frac{h\;\mathrm{lm}(v^{\text{‡}})}{k_{b}T}\right).$$

The rate of reaction was therefore calculated as the following equation:

2.17 $$k(T)=\chi (T)\times k^{\mathrm{TST}}(T).$$

In the Collins–Kimball theory [38], the apparent rate constant (*k*_app_) included the diffusion limit, which is necessary for the reactions close to or higher than the diffusion limit of the solution (equation (2.18)).

2.18 $$k_{\mathrm{app}}=\frac{k_{D}k}{k_{D}+k},$$

where *k* is the thermal rate constant and *k*_D_ is the steady-state Smoluchowski [39] rate constant for an irreversible bimolecular diffusion-controlled reaction.

Then, the overall rate constant (*k*_overall_) of the oxidation reaction of Leu by HO· was calculated according to equation (2.19) based on the molar factor of neutral form (*f_A_*) and dipole-salt form (*f_B_*):

2.19 $$k_{\mathrm{overall}}=f_{A}\times \sum k_{A}^{\mathrm{FHT}}+f_{A}\times k_{A}^{\mathrm{SET}}+f_{B}\times \sum k_{B}^{\mathrm{FHT}}+f_{B}\times k_{B}^{\mathrm{SET}}.$$

The branching ratio (*Γ*) characterizing the contribution of each reaction or pathway [21,40] was calculated as follows:

2.20 $$\Gamma_{i,j}=\frac{\;f_{i}\times k_{j}}{k_{\mathrm{overall}}}\times 100.$$

SEAGrid (https://seagrid.org) [41–44] is acknowledged for computational resources and services for the results presented in this publication.

## 3. Results and discussion

### 3.1. Structural and electronic properties

**Figure 2.**
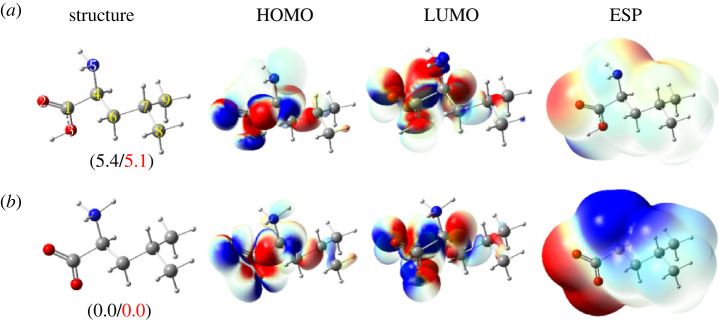
Optimized structures, HOMO–LUMO distribution and ESP maps (−0.064 to 0.064 a.u.) of l-leucine in water at M05-2X/6-311++G(d,p) level of theory (iso-value = 0.02) in two forms: neutral (*a*) and dipole-salt (*b*). The numbers in parentheses (in kcal mol^−1^) are the relative standard enthalpies (in black) and Gibbs free energies (in red) calculated at the M05-2X/6-311++G(3df,2dp)//M05-2X/6-311++G(d,p) level of theory.

Figure 2 illustrates optimized structures, HOMO, LUMO distribution and electrostatic potential (ESP) maps of l-leucine in water at the M05-2X/6-311++G(d,p) level of theory for neutral (*a*) and dipole-salt (*b*). Cartesian coordinates and the thermochemistry data of these forms are shown in electronic supplementary material, table SI1.

It is observed in figure 2 that the neutral form (*a*) contains –COOH and –NH_2_ functional groups, while the dipole-salt state (*b*) has –COO and –NH_3_ ones. HOMO and LUMO concentrate at O2, O3, C1, C4 and C6 atoms in both forms. Besides the N5 atom in structure (*a*) also has a large HOMO orbital. These areas are expected to be the interactive positions of Leu when it interacts with metal ions. Moreover, because of the existence of two polarized functional groups (i.e. COO and –NH_3_), the dipole-salt form of Leu is more polarized than the neutral one. Indeed, the negative regions in the ESP map of this form (in red colour) remarkably focus on two oxygen atoms, and the positive one (in blue colour) is significantly found in the –NH_3_ group. In addition, the dipole moment of form (*b*) (i.e. 15.3 Debye) is many times higher than that of form (*a*) (i.e. 2.1 Debye). As a result, the interactive ability with the polarized species (i.e. cations and anions) of the dipole-salt form is projected to be higher than that of the neutral one. Furthermore, the relative enthalpy and Gibbs free energy of dipole-salt form are 5.4 and 5.1 kcal mol^−1^ lower than those of neutral form, respectively. Consequently, the (*a*)-to-(*b*) transforming reaction has the stability constant (K*_f_*) being 5.64 × 10^3^ and thus, the dipole-salt form accounting for 99.98% in concentration, that is the main form of Leu in the biological environment.

### 3.2. Chelating ability of l-leucine

**Figure 3.**
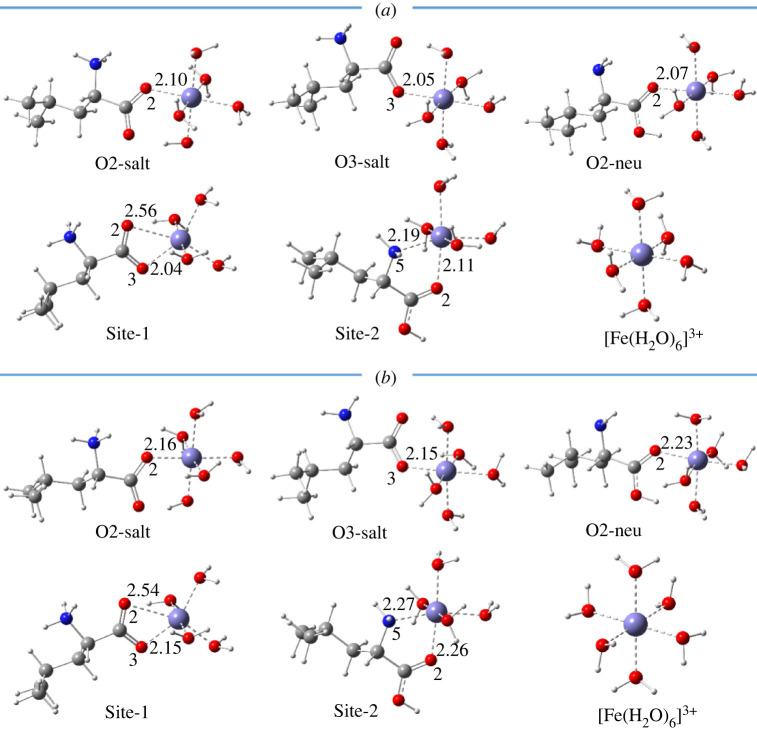
Optimized structures of five Leu complexes with 1 : 1 metal-to-ligand stoichiometric ratio at **O2-salt**, **O3-salt**, **O2-neu**, **site-1** and **site-2** chelating sites with (*a*) [Fe(H_2_O)_6_]^3+^ and (*b*) [Fe(H_2_O)_6_]^2+^ ions in aqueous phase. All distances are in Å.

**Figure 4.**
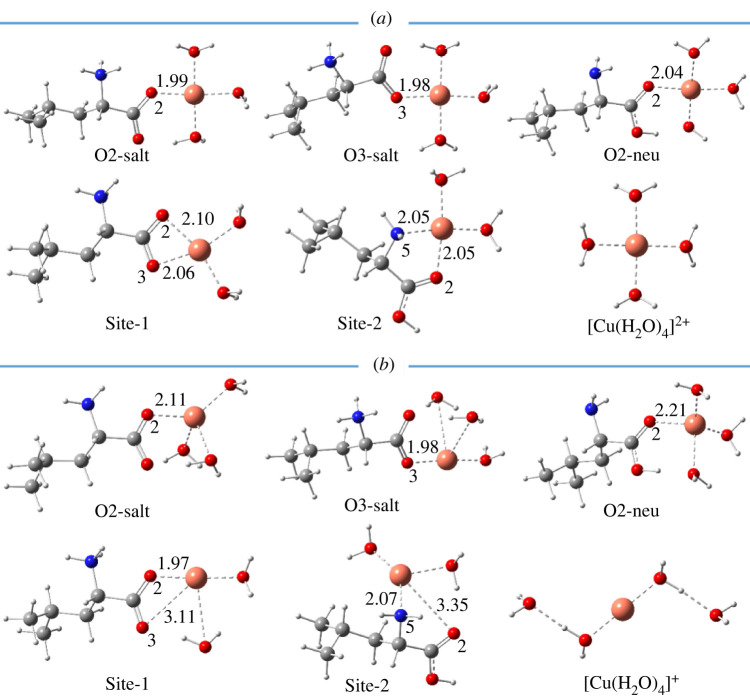
Optimized structures of five complexes with 1 : 1 metal-to-ligand stoichiometric ratio between Leu amino acid with hydrated ion (*a*) [Cu(H_2_O)_4_]^2+^ and (*b*) [Cu(H_2_O)_4_]^+^ ions at five chelating sites **O2-salt**, **O3-salt**, **O2-neu**, **site-1** and **site-2** in water. All distances are in Å.

Some previous works in the literature show that several organic compounds can form stable complexes with metal ions by their less stable existing form [24,45,46]; thus, the complexes of both neutral and dipole-salt forms of Leu are evaluated in this study. As shown in figure 1, Leu can trap metal ions via mono-dentate coordination at **O2-salt, O3-salt** and **O2-neu** sites, whereas the bi-dentate coordination occurs at two chelating sites named **Site-1** and **Site-2**. The optimized structures of the complexes between l-leucine (Leu) and hydrated Fe and Cu ions are presented in figures 3 and 4, whereas table 1 resumes Gibbs free energies (Δ_r_*G*^0^) and formation constants (*K*_f_) of the complexation reactions.

**Table 1.** The Gibbs free energies (Δ_r_*G*^0^, kcal mol^−1^) and formation constants (*K*_f_) of the complexation reactions between neutral and dipole-salt forms of Leu and Fe, Cu ions in aqueous phase calculated at the M05-2X/6-311++G(3df,2pd)//M05-2X/6-311++G(d,p) level of theory. The values in parentheses are the corresponding values calculated by the M05/6-311++G(3df,2pd)//M05/6-311++G(d,p) level of theory.

position	Fe(III) complexes		Fe(II) complexes		Cu(II) complexes		Cu(I) complexes	
	Δ_r_*G*^0^	*K_f_*	Δ_r_*G*^0^	*K_f_*	Δ_r_*G*^0^	*K_f_*	Δ_r_*G*^0^	*K_f_*
O2-salt	−7.8 (−8.5)	5.31 × 10^5^ (1.72 × 10^6^)	−3.0 (−3.7)	1.52 × 10^2^ (5.02 × 10^2^)	−6.9	1.13 × 10^5^	−1.8	2.06 × 10^1^
O3-salt	−5.6	1.36 × 10^4^	−2.7	1.01 × 10^2^	−7.2	1.76 × 10^5^	−2.8	1.04 × 10^2^
O2-neu	10.6	1.77 × 10^−8^	7.7	2.40 × 10^−6^	7.9	1.61 × 10^−6^	6.5	1.66 × 10^−5^
Site-1	−7.6	3.68 × 10^5^	−8.1	8.17 × 10^5^	−7.2 (−10.1)	1.92 × 10^5^ (2.47 × 10^7^)	−9.9 (−8.0)	1.69 × 10^7^ (7.25 × 10^5^)
Site-2	−0.3	1.53 × 10^0^	−2.0	2.90 × 10^1^	−5.8	1.85 × 10^4^	−6.9	1.06 × 10^5^

#### 3.2.1. Fe ions chelation

Figure 3 shows five structures of the complexes of Leu with ferric ion—[Fe(H_2_O)_6_]^3+^ and ferrous one—[Fe(H_2_O)_6_]^2+^. It can be seen that the interaction distances between Leu and Fe ions in mono-dentate complexes obtained at the **O2-salt**, **O3-salt** and **O2-neu** positions are shorter than those in bi-dentate complexes formed at the **Site-1** and **Site-2**. Indeed, the distances in the mono-dentate of Fe(III)-Leu and Fe(II)-Leu complexes vary from 2.05 to 2.10 Å and from 2.15 to 2.23 Å, respectively. Meanwhile, the ones in the bi-dentate complexes range from 2.11 to 2.56 Å and from 2.15 to 2.54 Å, respectively. As reported by previous works, the interaction distances in Fe(III) complexes are shorter than those in Fe(II) ones [24–27]. Cartesian coordinates and the thermochemistry data of all the Fe-Leu complexes are displayed in electronic supplementary material, table SI2.

In addition, Gibbs free energies (Δ_r_*G*^0^) and formation constants (*K*_f_) shown in table 1 indicate that most Fe-Leu complexes are stable, except the complexes of the neutral form at the **O2-neu** position. Indeed, the Δ_r_*G*^0^ values of the complexation reaction at **O2-salt**, **O3-salt**, **Site-1** and **Site-2** vary from −7.8 to −0.3 kcal mol^−1^ for Fe(III)-Leu complexes and from −8.1 to −2.0 kcal mol^−1^ for Fe(II)-Leu ones, and the corresponding *K*_f_ values vary from 1.53 × 10^0^ to 5.31 × 10^5^ and from 2.90 × 10^1^ to 8.17 × 10^5^, respectively. Furthermore, it is noteworthy that the **O2-salt** and **Site-1** are the most favourable chelating sites. The results of **O2-salt** complexes are compared with those calculated at the M05 method at the same basis set. We can see that the difference in Δ_r_*G*^0^ (kcal mol^−1^) between the two methods, M05-2X and M05, is not significant, being 0.7 kcal mol^−1^ for both Fe(III)- and Fe(II)-Leu complexes.

Overall, Leu in the dipole-salt form can favourably chelate [Fe(H_2_O)_6_]^3+^ and [Fe(H_2_O)_6_]^2+^ ions by forming stable complexes in which the Leu ligand mainly interacts with Fe ions through its –COO functional group (i.e. O2 and O3 atoms). Inversely, the chelating activity of the neutral form towards Fe ions is likely to be limited.

#### 3.2.2. Cu ions chelation

The optimized structures of five complexes between Leu amino acid with hydrated cupric ion [Cu(H_2_O)_4_]^2+^ (*a*) and cuprous one [Cu(H_2_O)_4_]^+^ (*b*) are presented in figure 4. The Cu(II)-O distances vary from 1.98 to 2.04 Å and from 2.05 to 2.10 Å for mono-dentate and bi-dentate complexes, respectively, whereas the Cu(II)-N distance is equal to 2.05 Å. Meanwhile, the Cu(I)-O distances in the mono-dentate Cu(I)-Leu complexes vary from 1.98 to 2.21 Å, while the Cu(I)-O2 (**site-1**) and Cu(I)-N5 (**site-2**) distances in the bi-dentate complexes are equal to 1.97 and 2.07 Å, the interaction distances O3-Cu(I) and O2-Cu(I) are remarkably longer being 3.11 and 3.35 Å. In addition, there is always one water molecule in these complexes making a hydrogen bond with O3 and O2 atoms of the Leu molecule. Cartesian coordinates and the thermochemistry data of all the Cu-Leu complexes are presented in electronic supplementary material, table SI3.

Similar to the Fe-Leu complexes, most of the reactions forming Cu-Leu complexes, except the ones at **O2-neu**, are spontaneous and favourable, with the negative Δ_r_*G*^0^ values varying from −7.2 to −5.8 kcal mol^−1^ for Cu(II)-Leu complexes and from −9.9 to −1.8 kcal mol^−1^ for Cu(I)-Leu ones (table 1). The formation constants (*K*_f_) of these reactions are therefore very high, especially one of the complexes at **site-1** being 1.92 × 10^5^ and 1.69 × 10^7^ for Cu(II)- and Cu(I)-Leu complexes. Nevertheless, the complexes at **O2-neu** of Cu(II) and Cu(I) ions are also dramatically less stable, with the Δ_r_*G*^0^ being 7.9 and 6.5 kcal mol^−1^, respectively. Consequently, the *K*_f_ values of these reactions are shallow (table 1). Furthermore, the results of the complexes at **O2-salt** are compared with those calculated at M05 functional with the same basis set. The difference in the Δ_r_G^0^ values between the methods M05-2X and M05 is also not remarkable, being 2.9 and 1.9 kcal mol^−1^ for Cu(II) and Cu(I) complexes, respectively.

Thus, Leu amino acid is expected to have a high potential to chelate both cupric and cuprous ions. The dipole-salt and neutral forms of Leu can interact with Cu ions via the –COO functional group and the **site-2** positions, respectively.

### 3.3. Pro-oxidant risks of l-leucine

The pro-oxidant risks of l-leucine are evaluated via the reduction reactions of Fe(III)-to-Fe(II)-Leu complexes and Cu(II)-to-Cu(I)-Leu complexes that may promote the production of reactive HO· radical by Fenton reactions [25,26,32]. Thermodynamic and kinetic data of the reduction reactions by two reducing agents, i.e. superoxide radical anion, O_2_·^–^, and ascorbate anion, Asc^–^, are shown in tables 2 and 3, respectively. The corresponding values for hydrated Fe(III)-to-Fe(II) and Cu(II)-to-Cu(I) ion reactions are used as references.

**Table 2.** Standard enthalpies (Δ_r_*H*^0^), Gibbs free energies (Δ_r_*G*^0^), nuclear reorganization (*λ*), Gibbs free energies of activation (Δ*G^‡^*), the diffusion rate constants (*k*_D_), thermal rate constants (*k*) and the apparent rate constants (*k*_app_) of reduction reactions of Fe(III)-to-Fe(II)-Leu complexes by superoxide radical anion (O_2_^·–^) and ascorbate anion (Asc^–^). Units of energy values and rate constants are in kcal mol^−1^ and M^−1^s^−1^, respectively.

position	Δ_r_*H*^0^	Δ_r_*G*^0^	*λ*	Δ*G^‡^*	*k_D_*	*k*	*k_app_*
[Fe(H_2_O)_6_]*^3+^* + O_2_^·–^ → [Fe(H_2_O)_6_]*^2+^* + O_2_; (R6)							
	−35.5	−39.5	22.6	3.2	7.62 × 10^9^	7.22 × 10^11^	7.54 × 10^9^
[Fe(H_2_O)_6-*n*_Leu]*^3+^* + O_2_^·–^ → [Fe(H_2_O)_6-*n*_Leu]*^2+^* + O_2_; (R8)							
O2-salt	−29.5	−34.6	20.3	2.6	8.10 × 10^9^	2.05 × 10^12^	8.07 × 10^9^
O3-salt	−32.4	−36.6	20.3	3.2	8.05 × 10^9^	6.35 × 10^11^	7.95 × 10^9^
O2-neu	−38.3	−42.4	23.7	3.7	7.92 × 10^9^	3.03 × 10^11^	7.71 × 10^9^
Site-1	−35.5	−39.9	20.8	4.4	7.98 × 10^9^	9.31 × 10^10^	7.35 × 10^9^
Site-2	−37.8	−41.2	21.1	4.8	7.90 × 10^9^	4.38 × 10^10^	6.69 × 10^9^
[Fe(H_2_O)_6_]*^3+^* + Asc^–^ → [Fe(H_2_O)_6_]*^2+^* + Asc^·^; (R7)							
	−8.8	−12.8	25.5	1.6	7.44 × 10^9^	1.06 × 10^13^	7.43 × 10^9^
[Fe(H_2_O)_6-*n*_Leu]*^3+^* + Asc^–^ → [Fe(H_2_O)_6-*n*_Leu]*^2+^* + Asc^·^; (R9)							
O2-salt	−2.7	−8.0	23.2	2.5	7.48 × 10^9^	2.26 × 10^12^	7.45 × 10^9^
O3-salt	−5.7	−9.9	23.2	1.9	7.46 × 10^9^	6.01 × 10^12^	7.46 × 10^9^
O2-neu	−11.6	−15.7	26.6	1.1	7.43 × 10^9^	2.31 × 10^13^	7.43 × 10^9^
Site-1	−8.7	−13.3	23.7	1.2	7.45 × 10^9^	2.17 × 10^13^	7.44 × 10^9^
Site-2	−11.1	−14.5	24.0	0.9	7.43 × 10^9^	3.19 × 10^13^	7.43 × 10^9^

**Table 3.** Standard enthalpies (Δ_r_*H*^0^) and Gibbs free energies (Δ_r_*G*^0^), nuclear reorganization (*λ*), Gibbs free energies of activation (Δ*G^‡^*), the diffusion rate constants (*k*_D_), thermal rate constants (*k*) and the apparent rate constants (*k_app_*) of reduction reactions of Cu(II)-to-Cu(I)-Leu complexes by superoxide radical anion (O_2_^·–^) and ascorbate anion (Asc^–^). Units of energy values and reaction constants are in kcal mol^−1^ and M^−1^s^−1^, respectively.

position	Δ_r_*H*^0^	Δ_r_*G*^0^	*λ*	Δ*G^‡^*	*k_D_*	*k*	*k_app_*
[Cu(H_2_O)_4_]*^2+^* + O_2_^·–^ → [Cu(H_2_O)_4_]*^+^* + O_2_; (R10)							
	−1.9	−5.9	29.6	4.8	7.58 × 10^9^	4.92 × 10^10^	6.57 × 10^9^
[Cu(H_2_O)_4-*n*_Leu]*^2+^* + O_2_^·–^ → [Cu(H_2_O)_4-*n*_Leu]*^+^* + O_2_; (R12)							
O2-salt	3.1	−0.8	28.6	6.8	8.07 × 10^9^	1.63 × 10^9^	1.36 × 10^9^
O3-salt	0.7	−1.5	29.5	6.7	7.89 × 10^9^	1.99 × 10^9^	1.59 × 10^9^
O2-neu	−2.9	−7.3	26.9	3.6	8.03 × 10^9^	3.61 × 10^11^	7.85 × 10^9^
Site-1	−6.4	−8.5	29.2	3.7	7.62 × 10^9^	3.21 × 10^11^	7.44 × 10^9^
Site-2	−3.6	−6.9	30.2	4.5	7.93 × 10^9^	7.77 × 10^10^	7.19 × 10^9^
[Cu(H_2_O)_4_]*^2+^* + Asc^–^ → [Cu(H_2_O)_4_]*^+^* + Asc^·^; (R11)							
	24.8	20.8	32.5	21.9	7.45 × 10^9^	1.43 × 10^−2^	1.43 × 10^−2^
[Cu(H_2_O)_4-*n*_Leu]*^2+^* + Asc^–^ → [Cu(H_2_O)_4-*n*_Leu]*^+^* + Asc^·^; (R13)							
O2-salt	29.8	25.9	31.5	26.2	7.47 × 10^9^	1.01 × 10^−5^	1.01 × 10^−5^
O3-salt	27.4	25.2	32.4	25.6	7.43 × 10^9^	2.56 × 10^−5^	2.56 × 10^−5^
O2-neu	23.9	19.4	29.8	20.3	7.46 × 10^9^	1.92 × 10^−1^	1.92 × 10^−1^
Site-1	20.3	18.2	32.1	19.7	7.44 × 10^9^	5.89 × 10^−1^	5.89 × 10^−1^
Site-2	23.2	19.8	33.1	21.1	7.44 × 10^9^	5.07 × 10^−2^	5.07 × 10^−2^

As observed in tables 2 and 3, the reduction reactions of Fe(III) to Fe(II) and Cu(II) to Cu(I) (i.e. R8/R9 and R12/R13) for the bi-dentate Leu (**Site-1** and **Site-2**) complexes and the **O2-neu** one with two reducing agents are all more favourable and exergonic than those of hydrated Fe(III)-to-Fe(II) and Cu(II)-to-Cu(I) ion ones (i.e. R6/R7 and R10/R11). Indeed, the Gibbs free energies of the reactions between Fe(III)-Leu complexes with O_2_·^–^ and Asc^–^ vary from −42.4 to −39.9 kcal mol^−1^ and from −15.7 to −13.3 kcal mol^−1^, respectively, which are more negative than that of hydrated Fe(III)-to-Fe(II) ion reactions being −39.5 kcal mol^−1^ for O_2_·^–^ and −12.8 kcal mol^−1^ for Asc^–^. Similarly, the Δ_r_*G*^0^ values for reduction reactions of Cu(II)-to-Cu(I)-Leu complexes (R12/R13) at **Site-1**, **Site-2** and **O2-neu** vary from −8.5 to −6.9 kcal mol^−1^ and 18.2 to 19.8 kcal mol^−1^ using O_2_·^–^ and Asc^–^ reducing agents, respectively, which are all lower than those of hydrated Cu(II)-to-Cu(I) ion reactions being −5.9 and 20.8 kcal mol^−1^, respectively. By contrast, the reduction reactions for the mono-dentate complexes of dipole-salt Leu (i.e. **O2-salt** and **O3-salt**) are less favourable than those of the hydrated metal ions. Indeed, the Δ_r_*G*^0^ values of the Fe(III)-to-Fe(II)-Leu complexes reactions with O_2_·^–^ and Asc^–^ are from 1.9 to 2.8 kcal mol^−1^ higher than that of hydrated Fe(III)-to-Fe(II) ion ones (table 2), while the Δ_r_*G*^0^ for Cu(II)-to-Cu(I)-Leu complexes reactions are 4.4 to 5.1 kcal mol^−1^ higher than that of hydrated Cu(II)-to-Cu(I) ion reactions (table 3).

Regarding the rate constants of the reduction reactions present in table 2, it is observed that the apparent rate *k_app_* values for the reaction of Fe(III)-to-Fe(II)-Leu complexes with O_2_·^–^ (from 6.69 × 10^9^ to 8.07 × 10^9^ M^−1^s^−1^) and Asc^–^ (from 7.43 × 10^9^ to 7.46 × 10^9^ M^−1^s^−1^) are very close to that of hydrated Fe(III)-to-Fe(II) ion reactions being 7.54 × 10^9^ and 7.43 × 10^9^ M^−1^s^−1^, respectively. As a result, the possibility of enhancing the formation of Fe(II)-Leu complexes that produce HO· radical via Fenton-like reaction is negligible. Regarding the reactions of Cu(II)-Leu complexes in table 3, it is noteworthy that the **Site-1**, **Site-2** and **O2-neu** complexes are expected to enhance the Cu(II)-to-Cu(I) reduction process. Indeed, the rate constants for the reduction reactions of Cu(II)-to-Cu(I)-Leu complexes by O_2_·^–^ agent vary from 7.19 × 10^9^ to 7.85 × 10^9^ M^−1^s^−1,^ which are more significant than that of hydrated Cu(II)-to-Cu(I) ion reaction being 6.57 × 10^9^ M^−1^s^−1^. Significantly, the *k*_app_ for the reactions of Cu(II)-to-Cu(I)-Leu complexes at these sites by Asc^–^ reducing agent varying from 5.07 × 10^−2^ and 5.89 × 10^−1^ M^−1^s^−1^ are 3.5 to 41 times higher than that of the hydrated Cu(II)-to-Cu(I) ion reaction (1.43 × 10^−2^ M^−1^s^−1^). Consequently, the pro-oxidant risks of these Cu(II)-Leu complexes are remarkable. By contrast, the reactions between the mono-dentate Cu(II)-Leu complexes of dipole-salt form with both O_2_·^–^ and Asc^–^ have lower *k*_app_ values than that of the [Cu(H_2_O)_4_]*^2+^* ion reactions. Therefore, these complexes do not enhance the Fenton reactions. It is noted that two bi-dentate complexes of Cu(II)-Leu are both stable, the reduction process forming [Cu(H_2_O)_4_]*^+^* ion from [Cu(H_2_O)_4_]*^2+^* can be enhanced, and therefore, this can promote the HO· radical formation via Fenton reactions. Notwithstanding, it is worth noting that Asc^–^ have HO· scavenging ability, which leads to the reduction of part of HO· radical produced by the pro-oxidant power of Leu.

In conclusion, the formation of the Fe(III)-Leu complexes does not enhance the Fenton reactions by the reduction reaction to Fe(II)-Leu complexes. However, the reduction reactions of stable Cu(II)-Leu complexes formed at **Site-1** and **Site-2** are favourable when ascorbate anion is present in the environment as a reducing agent.

### 3.4. Oxidation of l-leucine by hydroxyl radical

HO· radical is proved to be produced via the Fenton reactions initiated by the reduction reaction of Cu(II)-Leu complexes. The formed HO· radicals can then oxidize the Leu itself, damaging this amino acid. In this section, the oxidation mechanisms of Leu by HO· radical are therefore studied via the FHT and single-electron transfer (SET) reactions.

**Figure 5.**
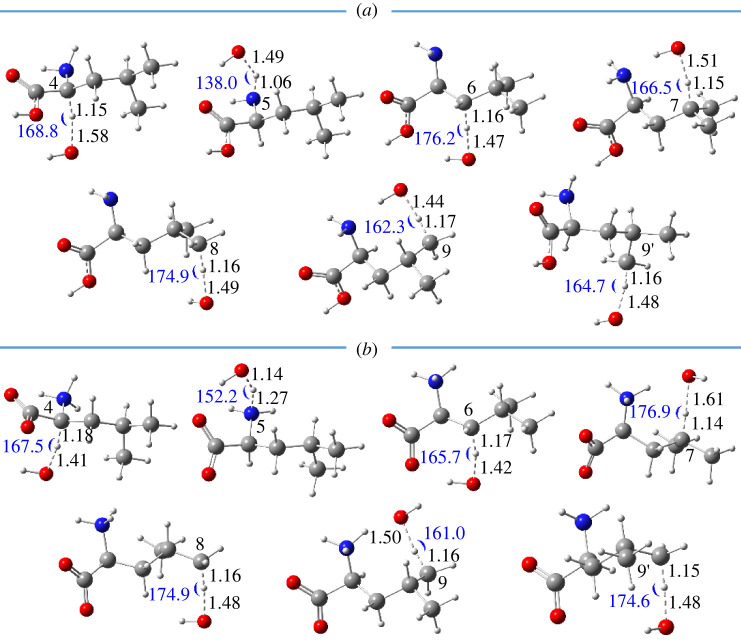
Optimized structures of the TSs of FHT reactions between HO· radical and l-leucine in the neutral form (*a*) and the dipole-salt form (*b*) in water. Distances (in black colour) are in angstrom (Å) and angles (in blue colour) are in degree (°).

The optimized structures of TSs for FHT reactions at seven sites of both neutral and dipole-salt forms are presented in figure 5. Two TS structures are recognized at the C9 atom, where the hydrogen bond between HO· radical and NH_2_/NH_3_ functional groups is observed and lowers its Gibbs free energy of activation. As seen in figure 5, the C/N···H bond distances at reactive sites vary from 1.06 to 1.18 Å, whereas the ones between the oxygen of HO· radical and the transferring hydrogen are from 1.41 to 1.58 Å. In addition, the interactive angles of the TSs, i.e. N-H-O or C-H-O, vary from 138.0° to 176.9°. Cartesian coordinates and thermochemistry data of these TSs are shown in electronic supplementary material, table SI4.

Table 4 resumes thermochemical and kinetic data for FHT and SET reactions between two existing forms of Leu and HO· radical. It is evident that most FHT reactions, except those at the N5-salt position, are favourable and spontaneous, with largely negative Δ_r_*G*^0^ varying from −42.1 to −14.2 kcal mol^−1^ and −20.5 to −13.7 kcal mol^−1^ for neutral and dipole-salt form, respectively. The most negative Δ_r_*G*^0^ for the FHT reactions of two Leu forms with HO· radical is found at the C4 position due to the formation of the hydrogen bond between the formed H_2_O molecular and the –COOH/–COO functional groups. The Δ*G*^‡^ values vary from 5.6 to 10.7 kcal mol^−1^ for FHT reactions of the neutral Leu and from 8.4 to 12 kcal mol^−1^ for the dipole-salt Leu. Furthermore, the rates of these reactions are all very high, being from 2.57 × 10^7^ to 2.71 × 10^9^ M^−1^s^−1^ for the neutral form and from 7.82 × 10^5^ to 5.97 × 10^8^ M^−1^s^−1^ for the dipole-salt one. Inversely, the reaction at the N5-salt position of the dipole-salt Leu is unfavourable, with high positive Δ_r_*G*^0^ and Δ*G*^‡^ values being 5.3 and 18.5 kcal mol^−1^, respectively. The rate of this reaction is also considerably slow being 5.78 × 10^4^ M^−1^s^−1^. Correspondingly, the total apparent rates of FHT reactions, *k*_app_^FHT^, for the neutral and dipole-salt forms are 3.65 × 10^9^ and 1.18 × 10^9^ M^−1^s^−1^, respectively. All the FHT reactions with HO· radical are in the diffusion limit. On the other hand, the SET reactions of Leu are unfavourable for both neutral and dipole-salt forms showing largely positive Δ_r_*G*^0^ (i.e. 12.9 and 26.7 kcal mol^−1^, respectively) and Δ*G*^‡^ values (13.8 and 39.3 kcal mol^−1^, respectively). As a result, the rates of the SET reactions of Leu are remarkably slower than those of FHT reactions. The apparent rate constant *k*_app_^SET^ of the neutral form is 1.20 × 10^4^ M^−1^s^−1^, whereas the one of the dipole-salt form is many times smaller being 2.49 × 10^−15^ M^−1^s^−1^. In addition, the overall rate constant, *k*_overall_*,* of the reactions between Leu and HO· is determined as 1.18 × 10^9^ M^−1^s^−1^.

**Table 4.** The Gibbs free energies (Δ_r_*G*^0^, kcal mol^−1^), Gibbs free energies of activation (Δ*G*^‡^, kcal mol^−1^), reaction path degeneracies (*σ*), tunnelling factors of Wigner (*χ*), diffusion rate constants (*k*_D_, M^−1^s^−1^), thermal rate constants (*k*, M^−1^s^−1^), apparent rate constants (*k*_app_, M^−1^s^−1^) and branching ratios (*Γ*, %) of the FHT and SET reactions between neutral and dipole-salt forms of l-leucine with HO· radical calculated at 298.15 K in water.

position	Δ_r_*G*^0^	ΔG^‡^	*σ*	*χ*	*k*_D_	*k*	*k*_app_	*Γ*
neutral form								
FHT								
C4-neu	−42.1	9.1	1	1.83	3.15 × 10^9^	5.82 × 10^7^	5.72 × 10^7^	0.00
N5-neu	−15.1	8.7	2	2.11	2.77 × 10^9^	2.99 × 10^8^	2.70 × 10^8^	0.00
C6-neu	−15.6	9.5	2	2.24	3.06 × 10^9^	7.63 × 10^7^	7.45 × 10^7^	0.00
C7-neu	−22.4	5.6	1	1.86	3.07 × 10^9^	2.34 × 10^10^	2.71 × 10^9^	0.04
C8-neu	−14.7	9.8	3	2.03	3.08 × 10^9^	5.56 × 10^7^	5.46 × 10^7^	0.00
C9-neu	−16.6	8.0	1	2.64	3.00 × 10^9^	5.37 × 10^8^	4.55 × 10^8^	0.01
C9′-neu	−14.2	10.7	2	2.03	3.04 × 10^9^	2.59 × 10^7^	2.57 × 10^7^	0.00
total	–	–	–	–	–	–	3.65 × 10^9^	0.05
SET								
	12.9	13.8	–	–	8.01 × 10^9^	1.20 × 10^4^	1.20 × 10^4^	0.00
dipole-salt form								
FHT								
C4-salt	−21.1	12.0	1	3.13	2.95 × 10^9^	7.82 × 10^5^	7.82 × 10^5^	0.07
N5-salt	5.3	18.5	3	5.78	2.69 × 10^9^	5.78 × 10^1^	5.78 × 10^1^	0.00
C6-salt	−17.3	8.4	2	2.55	2.96 × 10^9^	5.31 × 10^8^	4.50 × 10^8^	38.16
C7-salt	−20.5	7.35	1	1.18	3.15 × 10^9^	7.36 × 10^8^	5.97 × 10^8^	50.58
C8-salt	−13.7	10.0	3	2.04	3.02 × 10^9^	4.00 × 10^7^	3.95 × 10^7^	3.35
C9-salt	−16.2	9.2	1	2.16	3.01 × 10^9^	6.38 × 10^7^	6.25 × 10^7^	5.30
C9′-salt	−16.3	10.0	2	2.10	3.03 × 10^9^	2.94 × 10^7^	2.94 × 10^7^	2.49
total	–	–	–	–	–	–	1.18 × 10^9^	99.95
SET								
	26.7	39.3	–	–	7.94 × 10^9^	2.49 × 10^−15^	2.49 × 10^−15^	0.00
overall	–	–	–	–	–	–	1.18 × 10^9^	100.00

The branching ratios (*Γ*) present in table 4 illustrate that, due to the low percentage of the neutral form (i.e. 0.02%), the *Γ* values of the FHT reactions between the neutral state and HO· are from approximately 0.00 to 0.04% and the *Γ* total is only 0.05%. On the other hand, the values of dipole-salt form vary from 2.49 (C9′-salt) to 50.58% (C7-salt), except the *Γ* at N5-salt and C4-salt positions being 0.00 and 0.07%, respectively. The *Γ* total of these reactions is determined as 99.95%. In addition, the main product of the FHT reaction between Leu and HO· is found at C7-H position, accounting for more than half of the total products (*Γ* = 50.58%). By contrast, the *Γ* values of SET reactions of both the neutral and the dipole-salt are negligible, being approximately 0.00%.

In conclusion, the oxidation of Leu by HO· radical is favourable and spontaneous, with a sizeable overall rate constant being 1.18 × 10^9^ M^−1^s^−1^. In this process, the FHT reactions between dipole-salt form and HO· radical play a prominent role, accounting for 99.95%. Besides, the main FHT product of dipole-salt is at the C7-H position.

## 4. Conclusion

The Fe and Cu ions chelation activities of l-leucine (Leu) have been studied using the DFT approach in the aqueous phase. The possible reactive HO· radical formation initiated by the reduction reactions of Fe(III)-to-Fe(II)-Leu and Cu(II)-to-Cu(I)-Leu complexes was considered to evaluate the pro-oxidant risks enhanced by Fenton reactions. The oxidation mechanism of Leu by the formed HO· radical was also explored in detail. Multiple results are obtained as follows:

— Leu can spontaneously chelate Fe and Cu ions, especially at –COO functional group of dipole-salt form for both the mono-dentate and bi-dentate complexes.
— Fe(III)-Leu complexes exhibit negligible risk of pro-oxidant. However, Cu(II)-Leu complexes considerably enhance HO· radical formation initiated by the Cu(II)-to-Cu(I)-Leu reduction reactions, especially when the ascorbate anion is present in the reactive environment as a reducing agent.
— FHT reactions of dipole-salt mainly decide the oxidation process between Leu and HO· that occurs at multiple sites with the highest branching ratio found at the C7-H. As a result, the overall rate constant of this oxidation process is determined as 1.18 × 10^9^ M^−1^s^−1^.

Hopefully, the obtained results in this study shed more light on the chemical mechanism of the oxidation of l-leucine amino acid, which may be related to the chelation of transition metal ions.

## Data Availability

All relevant necessary data to reproduce all results in the paper are within the main text, electronic supplementary material [47] and the Dryad Digital Repository: https://doi.org/10.5061/dryad.vhhmgqnw5 [48].
